# Effectiveness of short message services and voice call interventions for antiretroviral therapy adherence and other outcomes: A systematic review and meta-analysis

**DOI:** 10.1371/journal.pone.0204091

**Published:** 2018-09-21

**Authors:** Isaac Amankwaa, Daniel Boateng, Dan Yedu Quansah, Cynthia Pomaa Akuoko, Catrin Evans

**Affiliations:** 1 Graduate School of Nursing, Midwifery & Health, Faculty of Health, Victoria University of Wellington, Wellington Regional Hospital, Wellington, New Zealand; 2 Julius Global Health, Julius Center for Health Sciences and Primary Care, University Medical Centre, Utrecht University, Utrecht, The Netherlands; 3 School of Public Health, Kwame Nkrumah University of Science and Technology, Kumasi, Ghana; 4 Service of Endocrinology, Diabetes and Metabolism, Lausanne University Hospital, Lausanne, Switzerland; 5 Department of Biomedical Sciences, University of Cape Coast, Cape Coast, Ghana; 6 Christian Service University College, Kumasi, Ghana; 7 School of Nursing, Queensland University of Technology, Brisbane, Australia; 8 School of Health Sciences, The University of Nottingham, Queen's Medical Centre, Nottingham, United Kingdom; Asociacion Civil Impacta Salud y Educacion, PERU

## Abstract

**Background:**

The potential of using mobile phone technologies to improve antiretroviral therapy (ART) adherence has provided a new facet to human immunodeficiency virus (HIV) research. The quality of evidence and the strength of recommendations of existing reviews, however, do not adequately support large-scale adoption of the intervention. This review adopted broad selection criteria to include all mobile phone-based interventions designed to improve patient’s adherence to ART.

**Methods:**

We performed a systematic review and meta-analysis of randomized controlled trials and quasi-experimental studies. PUBMED, MEDLINE, EMBASE, PsychINFO, Cochrane Central Register of Controlled Trials (CENTRAL), CINAHL, AMED and Web of Science were searched. Online abstracts archives of relevant conference proceedings and trial registries were also searched. Thirty-Five (35) full-text articles were assessed for eligibility. Included studies were conducted in high, low and middle-income countries and reported ART adherence interventions delivered by mobile phones (standard or smartphones) in the form of voice calls, interactive voice response calls (IVR), and short message service (SMS).

**Results:**

Thirteen (13) studies met the inclusion criteria, and 11 were used in the meta-analysis. Intervention characteristics of included studies ranged from mobile phone functionalities to provision of study phones to participants. SMS and voice call contents were tailored to participants’ specific adherence needs. Mobile SMS interventions improved adherence to ART compared with control conditions (OR, 95% CI = 1.59, 1.27–1.98). In subgroup analysis, only scheduled SMS was significant whereas triggered SMS had no effect on adherence to ART. Mobile voice calls did not significantly increase adherence to ART. The interventions were highly rated by > 90% of participants in the studies that reported on the experiences and satisfaction with the intervention.

**Conclusion:**

Scheduled mobile phone text-messaging have demonstrated significant improvement in adherence to ART. Mobile SMS adherence interventions that allow for two-way communication may, however, be more acceptable than standalone SMS reminders, which are seen to be intrusive, producing habituation and response fatigue. Voice calls and triggered SMS functionalities do not have a significant effect on adherence to ART although there is a higher preference for voice functionality over SMS especially in limited-resource and low-literacy settings. Further exploration of the mobile voice functionality and its possible combination with scheduled SMS functionality is recommended. Evidence provided in this study will guide the implementation of mobile phone intervention to improve adherence to ART, by addressing practical challenges that could militate against scalability especially in resource limited settings.

## Introduction

The introduction of Antiretroviral Therapy (ART) has led to a significant success in Human Immunodeficiency Virus (HIV) viral suppression, reduced the incidence of Acquired Immunodeficiency Virus (AIDS)-defining illnesses, and improved quality of life [1,2]. In recent times, ART has also been identified as a potent intervention to reduce the transmission of HIV among HIV serodiscordant couples [3–6]. Optimal adherence to ART has however emerged as a key determinant of ART’s success [7]. It is therefore desirable to provide adherence support for patients receiving ART to help preserve the effectiveness of first-line ART regimens.

Several approaches, ranging from the simplification of dosing schedules to the design of complex adherence interventions have been developed [8,9]. Among these interventions, mobile phones have emerged as a potentially more effective adherence support strategy [10] due to its increasing penetration rate, relatively low cost, convenience and the ability to offer varied communication approaches [11–13]. Mobile phone coverage is now near ubiquitous, with an estimated 95% of the global population living in an area covered by a basic 2G mobile-cellular network [14]. With documented evidence of its use in the management of several chronic conditions [15–17], mobile phones will be essential in facilitating the attainment of the 17 sustainable development goals (SDGs) [18].

Unfortunately, the quality of evidence and the strength of recommendations of existing studies and reviews do not support the widespread adoption of mobile phone interventions in ART adherence [19,20]. The recommendations from the two reviews (which included only RCTs and SMS-based interventions) may be limited to high literate populations who might already be familiar with sending and receiving text messages. The situation is summarised by two systematic reviews, which concluded that ‘mobile SMS is effective in improving adherence to ART [21,22]’. In the first systematic review, (carried out in 2012), Horvath and his colleagues [21] utilised the standard Cochrane review approach to identify studies utilising mobile phones for ART adherence. However, only two studies [23,24] met its strict inclusion criteria (only randomised controlled trials [RCT] utilising SMS messaging as an intervention). The authors found evidence of efficacy in using short weekly messages. The publication was followed by a recommendation by the World Health Organisation to consider text messaging ‘for promoting adherence to ART as part of adherence interventions [25,26].

Newer studies on SMS interventions have produced more contradictory results. The inconclusive findings, coupled with a need to determine the specific components of text-messaging trials that provided the observed effects, led to a second systematic review in 2014 by Finitsis et al., [22]. The eight RCTs that met the inclusion criteria found text-messaging interventions to be effective in supporting antiretroviral therapy adherence. Through sensitivity analyses, this review further reported on key design elements of the intervention that can potentially enhance the observed effects. These design elements include frequency of message delivery, directionality, tailoring of message content and matching the message to dosing timing. The literature so far has been inconclusive on these, and the authors recommended further exploration of these areas [22]

Despite the quality evidence generated by the two reviews, it remains unclear if the efficacies observed in these RCTs translates into effectiveness in broader and more realistic settings. While recommending RCTs to ascertain intervention’s effectiveness, attention could be paid to other forms of evidence (e.g. prospective cohort studies and pragmatic trials) that permit assessment of patients in their natural settings for longer periods.

Another real-world implementation dimension not addressed in the existing reviews is how SMS-based intervention may be implemented in low literacy populations. With emerging evidence suggesting that designs that encourage two-way communication may exhibit better outcomes [22], low literate populations may not benefit from the SMS-based intervention. A promising approach that may enhance engagement in this population is a phone call intervention. A preference for phone calls over text messages in populations with low-literacy and resource-constraint settings has been reported [27]. The limitations of existing SRs and the rate of incoming evidence calls for a more inclusive, up-to-date review that incorporates other forms of evidence. Our review adopts a broader scope to include mobile phone voice calls and non-RCTs. Findings of the current study will fill an essential gap in prior reviews that relied solely on SMS reminders and SMS-based interventions[23,28].

## Methods

The review was guided by methods developed by Joanna Briggs Institute [29,30] and followed the Preferred Reporting Items for Systematic Reviews and Meta-analysis (PRISMA) format [31].

### Search strategy

An initial limited search of major databases (PUBMED, CINAHL, MEDLINE and EMBASE) was undertaken to identify relevant keywords. This was followed by the development of a comprehensive and exhaustive search strategy, which was modified as appropriate across databases. The search strategy for PUBMED database is provided as a supplementary file (S1 Table).

The following electronic databases were subsequently searched in the period from January 1995 to June 2017: PUBMED, MEDLINE, EMBASE, Psych INFO, Cochrane Central Register of Controlled Trials (CENTRAL), CINAHL, AMED and Web of Science. Using different terms, trials that had been registered in the ‘ClinicalTrial.gov’ were also searched. Searches were limited to human studies but not by language or setting. The search strategy specified keywords, including ‘text messaging’, ‘SMS’, ‘voice calls’, and ‘phone calls’ combined with ‘adherence or compliance’. We also searched online abstract archives of relevant conference proceedings, including the conference on Retroviruses and Opportunistic Infections (CROI), International AIDS Conference (IAC), and the International AIDS Society (IAS) Conference on HIV Pathogenesis, Treatment and Prevention. Finally, a hand-search of the reference lists and bibliographies of potential studies was performed.

### Study selection

Electronic search results were imported into Endnote X7 bibliographic software [32] and duplicates removed by one author (IA). This was followed by an independent screening of titles and abstracts against set inclusion and exclusion criteria by IA, DB and DYQ. Two authors (IA and DB) independently screened full texts of all potentially relevant studies. Disagreements were resolved by consensus or discussion by all authors.

### Eligibility criteria

Papers eligible for inclusion were informed by inclusion and exclusion criteria applied to each of the following domains: study types, types of participants, types of interventions and types of outcomes. The inclusion and exclusion criteria within each of the domains are described below:

Study types: We included randomised controlled trials (RCTs) and quasi-experimental studies that examined the effectiveness of mobile phone-based interventions for ART adherence. Qualitative only designs, letters to editors and commentaries on published studies were excluded.

#### Types of participants (P)

This review included HIV positive individuals receiving ART, irrespective of age (to include carers in the case of vulnerable adults or children). Further, studies were included irrespective of gender or clinical stage of HIV infection in a primary, outpatient, community and hospital care settings.

#### Types of interventions (I)

Interventions of interest were voice calls, short message service (SMS) and Interactive Voice Response Calls (IVR) that were delivered by mobile phones (standard or smartphones) and targeted ART adherence. We defined IVR as an automated telephony system that interacts with callers, through mobile phones, gathers data and directs calls to the appropriate recipient [33]. Studies in which adherence interventions were augmented by smartphone applications (apps) or delivered to fixed telephone landlines were excluded.

#### Comparator (C)

Mobile phone-based interventions were compared with existing standard adherence interventions such as self-monitoring, reinforcement, counselling, and psychological therapy [34]. Studies were excluded if the comparator is also an adherence reminder such as an alarm system.

#### Types of outcomes (O)

The primary outcome was ART adherence. This was measured directly (e.g. pill count) or indirectly (e.g. self-reporting), and secondary outcomes were HIV viral load (quantity of virus in a given volume, copies/mL), quality of life and patient satisfaction with the mobile phone intervention.

### Data extraction

A data extraction sheet based on Joanna Briggs Institute (JBI) data extraction forms [35] was developed, pilot-tested on four (4) randomly selected included studies and refined as appropriate. Details of the included studies were then extracted by one reviewer (IA), and checked by a second reviewer (DB). Disagreements were resolved with a third reviewer (DYQ). Data extracted from each study included study setting, the type of mobile technology employed, study size, gender, age of participants, ART treatment experience, mobile phone ownership and adherence status/viral load. We also included how message content was developed, duration of the study, delivery frequency, main outcome(s) measured, methods of outcome assessment and major findings.

### Quality assessment and risk of bias across studies

The JBI critical appraisal checklist [36], (one each for included RCTs and experimental/cohort studies), comprising a list of questions with fixed responses, were used to assess the quality of included studies. Two authors independently graded all included articles, and a senior author (CE) reviewed all the grades. Each article was scored on certain intervention characteristics (e.g. randomisation, blinding) [29]. Any disagreements were resolved between the authors in a scheduled meeting. The quality assessment and scoring criteria are available in Tables A and B in S2 File.

### Data synthesis and analysis

Intervention studies that provided sufficient data were used in separate meta-analyses using Cochrane Review Manager (RevMan)[37] software. Findings of the remaining studies were presented in a narrative format.

We focused on dichotomous outcomes and the odds ratios and 95% confidence interval (CI) for the effect of mobile phone interventions in adherence to ART. Using a formula by Chinn [38], the effects measures that were on a continuous scale were re-expressed as log odds ratios using standardized mean difference (SMD). The studies that reported odds ratios were also converted to log odds ratios. The log odds ratios and standard errors (SE) were combined in RevMan using the generic inverse-variance method [39]. Sub-group analyses were done for scheduled and triggered SMS. The overall effect of mobile phone intervention on immunological and virological outcomes was also estimated using the pooled odds ratio for viral load <400copies/ml [40]. We performed a fixed effect analysis, and heterogeneity was assessed using the Cochrane’s Q and degree of inconsistency (*I*^*2*^) [41]. A sensitivity analysis, however, showed little difference in estimates in the fixed or random effect model. Odds ratios were considered statistically significant at the 5% level (P<0.05) if the value 1 is not within the 95% CI.

## Results

### Characteristics of included studies

**Fig 1:**
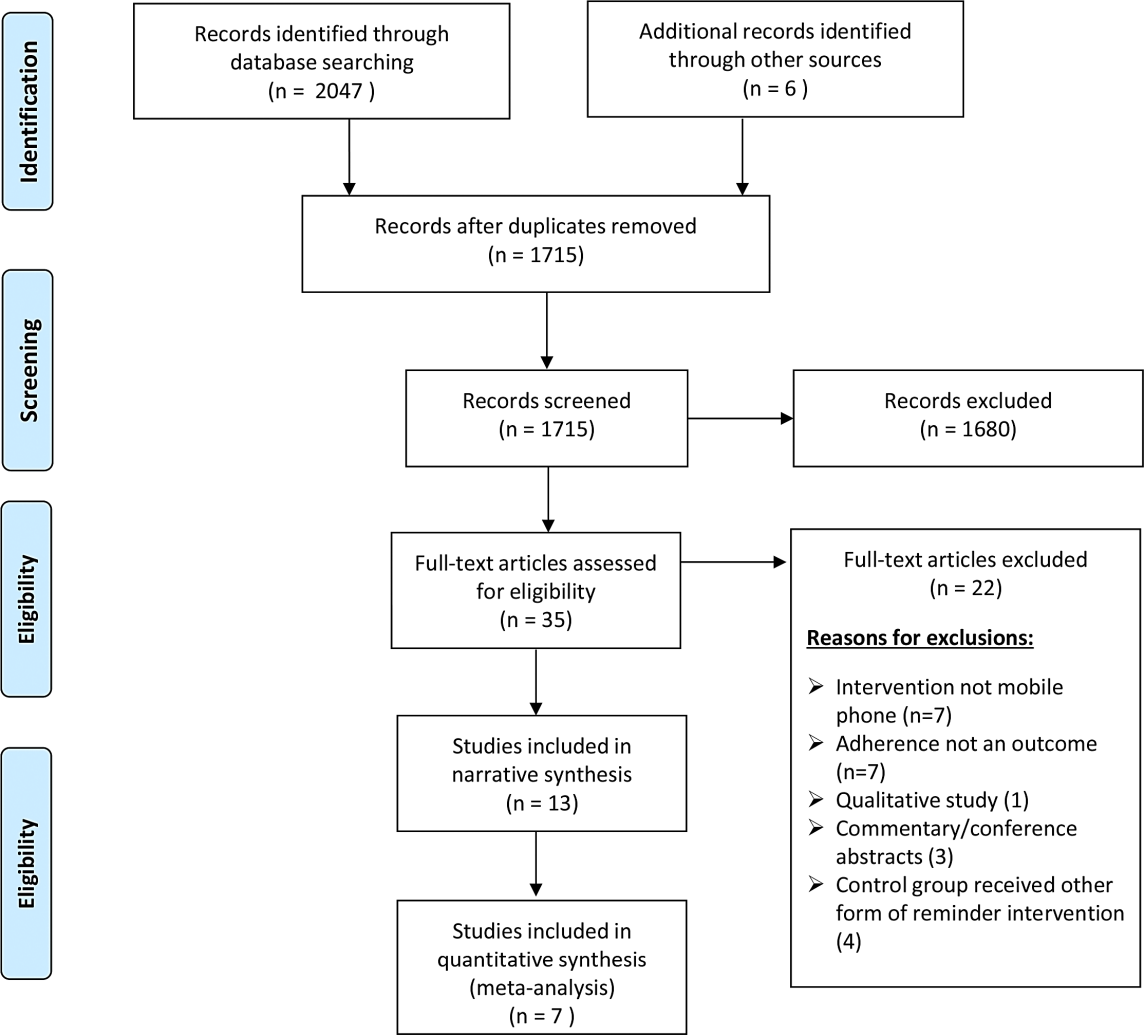
Flow chart of inclusion and exclusion criteria of relevant articles.

Thirteen (13) RCTs and 2 cohort and quasi-experimental studies met the inclusion criteria with 22 excluded with reasons, Fig 1.

Table 1 summarizes the characteristics of the included studies. Studies were conducted in high- (USA, n = 2; Italy, n = 1), low- and middle-income countries (Kenya, n = 2; India, n = 2; Brazil, n = 1; China, n = 2; Nigeria, n = 1, Cameroon, n = 1, Uganda, n = 1), at Infectious Disease and ART clinics (n = 4), health care clinics (n = 2), community health centres (n = 2), hospitals (n = 4) and reported between 2010 and 2016. Sample sizes ranged from 21 to 631.

**Table 1:** Characteristics of included studies and participants.

Author (year)	Country	Setting	Study size	Gender		Age of participants (years)	ART treatment experience	Mobile phone ownership	Adherence status/viral load
				M	F				
***Randomized control trials***									
Lester et al. (2010b)[23]	Kenya	Healthcare clinic	528	-	-	>18	Less than 3 months on ART	Personal phone	Not reported
Kalichman et al. (2011)[45]	USA	Infectious disease clinic	40	26	14	>18	Receiving ART (duration unspecified	Study phone provided	Sub-optimal adherence
Pop-Eleches et al. (2011)[24]	Kenya	Rural health centre	428	-	-	>18	Less than three months on ART	Study phone provided	Not reported
da Costa et al. (2012)[42]	Brazil	Health centre for infectious diseases	25	-	21	Not specified	Less than three months on ART	Personal phone	CD4 count >200/mm^3^
Mbuagbaw et al (2012)[47]	Cameroon	Central hospital	200	-	-	>21	Been on ART for at least 1 month	Personal phone	Not reported
Huang et al. (2013)[52]	China	3 county hospitals	196	94	102	≥18	Treatment-naïve and Treatment-experienced	Personal phone	Baseline CD4 count < 350 cells/mm^3^
Shet et al. (2014)[50]	India	Health care clinic	631	358	273	18–60	ART naïve	Study phone provided	Sub-optimal adherence
Maduka and Tobin-West (2015)[48]	Nigeria	Tertiary health care hospital)	104	45	59	Mean age [control: 36; intervention:37]	Treatment Experienced	Personal phone	Sub-optimal adherence
Belzer et al. (2015)[51]	USA	Not specified	37	23	14	15–24	Unspecified	Personal and study phones	Sub-optimal adherence
Sabin et al. (2015)[46]	China	ART clinic in Nanning	120	-	-	>18 (Mean age = 38)	Treatment experience and no experienced	Personal phone	Sup optimal adherence
Haberer et al (2016)[49]	Uganda	Mbarara regional referral hospital	63	23	40	>18	Individuals initiating ART	Personal phone	Not reported
***Quasi–experimental time series/Prospective studies***									
Rodrigues et al. (2012)[53]	India	Infectious disease clinic	150	109	41	Mean age = 38	On ART for at least a month	Personal phone	Not reported
Dowshen et al. (2012)[43]	USA	Community-based health centre	25	23	2	14–29	Receiving ART (duration unspecified)	Personal phone	Sub-optimal adherence

Majority of the studies recruited adult participants (> 18 years) of both sexes (54.63% male). Three studies focused their interventions on specific populations such as Latina women in Brazil [42] and the youth (14–29 years) [43]. Two studies [43–45] recruited only participants with established suboptimal adherence. Studies considered participants who were receiving or initiating ART [46], had been on ART for at least one month [47], or for less than three months [23,24,48,49]. Shet et al., [50] recruited HIV positive individuals who were ART-naive and had adequate documentation on their HIV positive status. Belzer et al., [51] focused on documented HIV positive patients with a history of non-adherence to one or more components of ART. Huang et al., [52] on the other hand recruited both treatment-naive and treatment-experienced HIV positive patients. The most commonly cited inclusion criteria were age (9/13), followed by access to personal (or shared) phone (8/13). One study [48] removed participants with chronic disease necessitating daily medication while another [51] removed participants with cognitive impairment or substance abuse. Illiteracy was an exclusion criterion for one study [42].

### Risk of bias

The RCTs employed adequate randomisation techniques, had groups that were similar at baseline and measured outcomes of the two groups in a similar and reliable way. Appropriate allocation concealment was adopted by all the trials except Pop-Eleches et al. [24], who failed to report on this. Understandably, blinding of participants to treatment allocation was a point missing in all studies since the intervention required overt participation. Risk of contamination was a concern in the study by Lester et al., [23] as several participants were reported to have forwarded their weekly text messages to non-intervention participants to share support. In the case of Kalichman et al., [45] bi-weekly calls to the control group was made by an assessment staff whereas the intervention group received calls from a counsellor who was a master’s level social worker with experience in AIDS services. The study further mailed supportive devices like an alarm clock and post-it reminders to participants in the intervention group who identified these items as adherent needs. The above constitute the provision of treatments other than the intervention of interest and might have caused a systematic difference; thereby risking skewed results, Table A S2 File.

The cohort/quasi-experimental studies instituted measures that ensured that samples were representative of the population, minimised selection bias, the reliability of measured outcome and conducted appropriate statistical analysis, Table B in S2 File. An element of selection bias might have been introduced in most of the included studies as the authors excluded participants who had no access to personal mobile phones.

### Description of interventions

Studies varied in mobile phone functionality, content and delivery of the intervention (Table 2).

**Table 2:** Characteristics of interventions and outcome measurements.

Author (year)	Mobile technology	Nature of message delivered	Duration (months)	Delivery frequency	Outcome measured	Outcome
***Randomized control trials***						
Lester et al. (2010b)	SMS check-ins	*Intervention*: SMS check-ins *Control*: Usual care	12	Weekly	ART adherence measured by self-report HIV-1 Viral suppression, Total attrition	**Adherence outcome** Adherence to ART was reported in 168 of 273 patients receiving the SMS intervention compared with 132 of 265 in the control group (relative risk[RR] for non-adherence 0.81, 95% CI 0.69–0.94). **Viral load** Suppressed viral loads were reported in 156 of 273 patients in the SMS group and 128 of 265 in the control group, (RR for virologic failure 0.84, 95% CI 0.71–0.99)
Kalichman et al. (2011)	Voice	*Intervention*: Behavioral self-regulation counselling phone call. *Control*: Usual care.	4	4-biweekly calls	ART adherence Self–efficacy Adherence measured by Pill count and response to a challenge situation	**Adherence outcome** Adherence improved from 87% of pill taken at baseline to 94% adherence 4 months after baseline in the intervention group compared with **Self-efficacy** Behavioural self-management counselling condition demonstrated greater self-efficacy for medication adherence.
Pop-Eleches et al. (2011)	SMS	*Intervention*: Short and long simple messages developed in conjunction with clinic staff. Participants specified their preferred languages. *Control*: Usual care	12	Daily or weekly interval	Adherence and treatment interruptions measured by MEMS	**Adherence outcome** 53% of participants receiving weekly SMS reminders achieved adherence of at least 90% during the 48wks of study, compared with 40% of participants in the control group. **Treatment Interruptions** Participants in groups receiving weekly reminders were also significantly less likely to experience treatment interruptions exceeding 48h during the 48-wk. follow-up period than participants in the control group (81 vs 90%)
da Costa et al. (2012)	SMS	Intervention: The message ‘take good care of your Health’ was chosen by the multidisciplinary team involved in patient care and researchers. *Control*: Usual care	12	Weekends and alternate days during the week	Adherence >95% (1^st^ to 4^th^ month) measured by: Self-reported Pill counting MEMS	**Adherence outcomes** Self-reported: 11 participants (84.62%) remained adherent in the control group vs.8 participants (100%) in the intervention group Pill counts: 5 participants (38.46%) for the control group and 4 (50.00%) in the intervention group remained compliant MEMS: 6 participants (46.15%)in the control group and 6 participants (75.00%) in the intervention group remained compliant
Shet et al. (2014)	Voice call	*Intervention*: Customized motivational voice call reminders and a pictorial message. Patients chose the sex and language of the pre-recorded message *Control*: Usual care	24	Once a week and a weekly reminder after four days of call	Adherence measured by pill count	**Adherence outcome** Comparison of suboptimal adherence was similar between both groups (unadjusted incidence rate ration 1.24, 95% CI 0.93 to 1.65, P = 0.14). Incidence proportion of patients with suboptimal adherence was 81/300 (27%) in the intervention arm and 65/299 (21.7% in the standard arm
Maduka and Tobin-West (2015)	SMS	*Intervention*: Adherence counselling and short message reminders *Control*: Usual care	4	Twice a week	ART adherence measured by self-report CD4 count	**Adherence outcome** At post-intervention, 76.9% of the intervention group and 55.8% of the control group achieved adherence (χ^2^ = 5.211, P = 0.022, RR = 0.75 (0.55–0.96). Median CD4+ cell count of the intervention group increased from 193 cells/ml to 575.0 cells/ml against 131.0cells/ml to 361.5 cells/ml in the control group (P = 0.007)
Belzer et al. (2015)	Voice call	*Intervention*: Customized calls with a conversation that included medication review, problem-solving support and scheduling of relevant referrals. *Control*: usual care	6	Weekly	ART adherence measured by self-report Viral loads	**Adherence outcome** Adherence higher in the intervention group compared to the control group (P = 0.007) **Viral load** Log 10 HIV VL was significantly lower at 24 weeks (2.82 vs 4.52, P = 0.002) and 48 weeks (3.23 vs 4.23, P = 0.043)
Sabin et al. (2015)	SMS	*Intervention*: Adherence counselling and SMS phone reminder when Wisepill system fails to open 30minutes after scheduled dose time *Control*: usual care	9	30 mins after wise pills fail to detect device opening	ART adherence Viral loads UDVL rates	**Adherence outcome** Mean adherence of 96.2% vs 89.1% (p = 0.003) was recorded for the intervention and control respectively. ***Suboptimal adherers*:** Mean adherence of 93.3% vs 84.7% (p = 0.039) for the intervention and control groups respectively were recorded. ***Optimal adherers*:** Mean adherence of 97.8% vs 91.7% (p = 0.028) for the intervention and control groups respectively.
Huang et al. (2013)	Voice calls	Intervention: mobile phone conversations consisted of semi-structured dialogue eliciting the reasons and difficulties in making a hospital visit, symptoms and treatment, treatment adherence and difficulty in taking medications. *Control*: Usual care	3 months	reminder calls made every two weeks	Adherence measured by self-report Viral load Quality of life measured by WHOQOL-HIV-BREF	**Adherence outcome** **Treatment-naïve:** mean adherence of 99.7 (SD-1.6) and 96.5(SD-17.8) for the intervention and control groups respectively (p = 0.09). **Treatment-experienced:** mean adherence of 99.6 (SD-1.0) and 99.5 (SD-2.0) for the intervention and control groups respectively(p = 0.37). **CD4 (cell count/mm**^**3**^ **)** Overall viral load significantly decreased, and CD4 count significantly increased from baseline to follow-up. **Quality of life** After 3 months, significant QOL improvements were observed in domains of physical health (p = 0.003), level of independence (p = 0.018), environment (p = 0.002), and spirituality/religion/personal beliefs (p = 0.021) among treatment-naïve patient
Mbuagaw et al. (2012)	SMS	*Intervention*: motivational messages with reminder component and a phone number that patient could call back if they needed help. The content was varied and contemporary. There were 11 messages that were changed weekly *Control*: Usual care	6	Once every Wednesday at 9:00 am	Adherence to ART measured by Visual analogue scale (VAS) Quality of life (QOL)	**Adherence outcome** No significant effect on adherence was found by: - VAS>95% (risk ratio [RR] 1.06, 95% [CI] 0.89, 1.29; - reported missed doses (RR 1.01, 95% CI 0.87, 1.16; -number of pharmacy refills (mean difference [MD] 0.1, 95% CI: 0.23, 0.43
Haberer et al.(2016)	SMS reminder	*Intervention* Scheduled SMS arm: Scheduled SMS + real-time adherence monitoring Triggered SMS arm: Triggered SMS + real-time adherence monitoring *Control* Real-time adherence monitoring only Study participants in this arm received no SMS reminders.	9	Scheduled: daily SMS for 1 month, weekly for 2 months Triggered: SMS received only if no signal is received in 2 hrs	Adherence measured by Wise pill monitoring technology HIV RNA suppression	**Adherence outcome** Compared to control, adherence was 11.1% higher (P = 0.04), and more than 48-h lapses were less frequent (IRR 0.6, P = 0.02) in the scheduled SMS arm. Adherence and more than 48-lapses were similar in the triggered SMS arm and control **HIV RNA suppression** No difference in HIV RNA was seen
***Quasi–experimental time series/ prospective studies***						
Rodrigues et al. (2012)	Interactive voice response (IVR) Non- interactive neutral picture SMS	Two reminders (interactive calls and non-interactive neutral short SMS)	6	Weekly	ART adherence measured by pill count	**Adherence outcome** The proportion of participants with optimal adherence increased from 85% to 91% during the intervention period, an effect that was maintained 6 months after the intervention was discontinued
Dowshen et al. (2012)	SMS	Personalized SMS reminders	6	Daily	Adherence measured by Visual analogue scale CD4 cell count Viral load monitoring	**Adherence outcomes** Participants increased from a baseline value of 74.7 on the VAS to 93.1 at the 24-week follow-ups. **Viral load suppression** No significant difference in CD4 cell count or viral load between baseline and 12- or 24-week follow-up

SMS, short message service; ART, antiretroviral therapy

MEMS, Medication event monitoring system

UDVL, Undetected viral load levels

WHOQOL-HIV BREF, World Health Organization Quality of Life in HIV-infected Person instrument

*VAS*, *Visual analogue scale*

#### Mobile phone technologies

***Mobile phone functionalities*** employed by the included studies were SMS reminders (8/13), voice (4/13) and a combination of SMS and Interactive Voice Response calls (1/13). Sabin et al., [46] and Haberer et al., [49] adopted an electronic adherence-monitoring device, which trigger an SMS reminder message to be sent whenever the Wisepill system (a pillbox with a Global system for mobile [GSM] communication that makes use of mobile phone and internet technology to provide real-time medication management) failed to detect a device opening 30 minutes after scheduled dose time.

In other studies, participants who had no access to personal phones were provided with study phones [24,45,50,51], excluded (10/13) or made to share phones with close relations [23]. Haberer et al., [49] excluded 49 (37%) of potential study participants because they had no personal phones.

***The cost of the entire intervention*** was not reported by any of the reviewed trials. An average amount of US$ 0.08 was however reported as the cost of sending an SMS by three studies [23,42,47]. One study reported a monthly $45.00 cell phone plan for participants who chose to use their phones or those who were given mobile phones [51]. Such mobile phone plans restricted participants from making toll calls, downloading applications and any other functions that would add additional costs. Incentives in the form of cash [24,43,46] and phone credit [24] were also reported. Before the intervention, 4 out of 13 studies trained participants on how to respond to incoming interventions but failed to report on the cost of this training. [23,24,45,53].

#### Message development, content and tailoring

***The processes involved in developing messages*** were described in 7 out of 13 studies. Three of these [23,24,42] involved a multidisciplinary team and study participants in message content development. Dowshen et al., [43] for example collaborated with patients to develop their messages; Sabin et al., [46] provided patients with the option of selecting from a list 10 options developed jointly by clinicians and subjects. Lewis et al., [54] on the other hand employed the services of three experts with extensive experience in HIV interventions for men who have sex with men (MSM). The content of messages was constructed to remind (8/13), support (6/13) or counsel (1/13) participants. In the case of Belzer et al., [51], adherence facilitators were recruited and together with participants, scheduled call times at the convenience of participants.

***Evidence of tailoring content of messages*** to participant’s specific adherence needs was seen in only 7 studies [43,45,50–54]. These studies either supported patients to design their messages [43] or depended on feedback/specific answers provided by patients to adherence questions. Huang et al., [52], Kalichman et al., [45] and Lewis et al. [54] for example delivered tailored messages that varied in content and frequency based on participants’ experience with ART regimen. In other studies, participants also chose the sex and language of a pre-recorded voice call to be delivered [50] and the language of the voice call to be made [53] In the case of Belzer and colleagues[51], participants who reported being treatment-naive/non-adherent received daily messages (1–3 times daily) whereas therapeutically adherent participants received weekly messages. Also, participants were given a chance to change their call times during the study period for their convenience in consultation with adherence facilitators [51].

***Theoretical framework*** utilised in developing messages was made evident in two (2) studies. Kalichman et al. [45], relied on self-regulation models of medication adherence and offered corrective feedbacks that directly addressed the identified adherence needs of participants whereas Mbuagbaw et al., [47] utilised the health behaviour model of behaviour change. All included studies showed ***evidence of ensuring privacy and confidentiality of HIV status*** of the participant as depicted by messages being generally brief and neutral with no mention of ‘HIV’, ‘AIDS’, or ‘ART’. A participant withdrew from a study [47] due to privacy concerns. Six out of 286 participants from the Shet et al., [50] study expressed concerns about intrusiveness and loss of privacy at the end of the follow-up period of intervention.

#### Delivery strategies

***An automated message delivery function*** was employed by five (5) studies to send SMS messages to participants. Three (3) studies [42–44,46] further designed a study specific, web-based interface to ensure efficient management of patient information and text messaging. One study [48], made use of an internet-based bulk SMS platform to send out pre-scripted messages to participants. There was also an automatic voice call delivery function employed by Shet et al., [50] which began by greeting participants with a hope message before inquiring if patients had taken their medications.

***Frequency and timing of communication*** varied within and across studies. Messages were delivered on a daily [43,44,51], alternate days [42], weekly [23,50], twice a week [48], every two weeks [45,52] and a combination of daily and weekly [24]. Delivery of SMS messages/voice calls occurred close to or at the time of dosing [42,44], at fixed times in the day [23,24,47], or as specified by the participant [43,51]. Two-W***ay interactivity*** between study participants and investigators was evident in seven (7) studies [23,43,45,47,50–52]. These studies made it possible for message recipients to message back and forth with clinic staff. These participants had the opportunity to respond to a specific cue, report a problem or ask questions regarding their care. A total of 15598 valid responses to SMS inquiries were received from 815 participants in 3 studies [23,43,47] that required some form of feedback. Reasons for responding were mainly to request medical, logistical, financial support and/or to express gratitude. In all the studies, calls and/or text messaging were initiated by the study investigator. The length and content of messages varied in most studies reviewed. Pop-eleches et al., [24] for example used a combination of long and short messages whereas Mbuagbaw et al., [47] designed a series of 11 messages that were changed every week. This variation in content and length of messages were to sustain interest, provide encouraging content and prevented habituation.

### Effects of mobile phone-based interventions on ART

#### Primary outcome: ART adherence

Characteristics of outcomes are summarised in Table 2. Adherence to ART was measured as a primary outcome in all included studies. Methods and time intervals employed by the various studies in measuring adherence varied across studies.

Eleven (11) RCTs were included in the meta-analysis. The other two quasi-experimental or prospective cohort and had different scales of measurement of the outcome and therefore could not be pulled together with the RCTs. Seven (7) of these measured the effect of SMS intervention [23,24,42,46–49] whereas four (4) measured the effect of voice calls [45,50–52]. Haberer et al., [49] had two SMS intervention arms, and these were used separately in the subgroup analysis.

**Fig 2:**
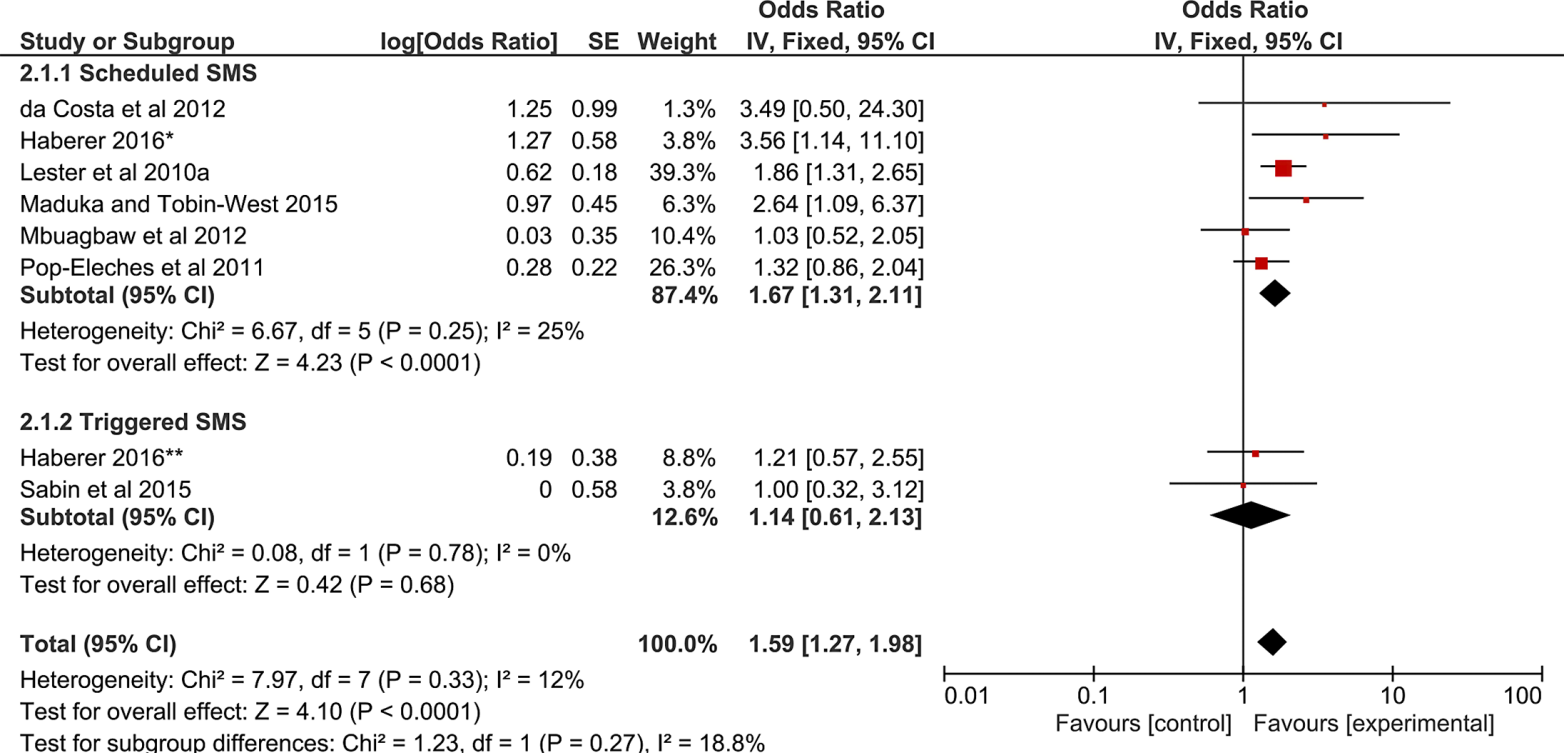
Forest plot of pooled odds ratio and 95% CI for effect of SMS intervention on adherence to ART. *Scheduled SMS arm, **triggered SMS.

Mobile SMS intervention had a significant effect on adherence to ART (OR, 95% CI;1.59;1.27–1.98), Fig 2.

**Fig 3:**
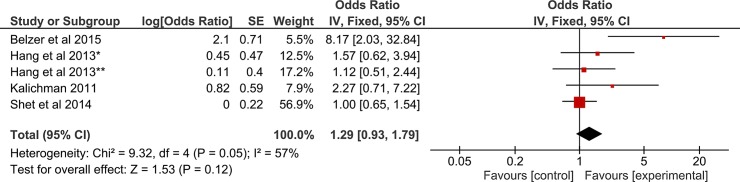
Forest plot of pooled odds ratio and 95% CI for effect of voice call intervention on adherence to ART. *ART naïve group, **ART experienced group.

In the subgroups, however, only scheduled SMS significantly increased adherence to ART. Mobile voice interventions had no significant association with adherence to ART, Fig 3.

#### Quasi-experimental studies

The two (2) non-randomised trials also reported beneficial adherence outcomes. Dowshen et al. [43] demonstrated that personalised, daily text messages delivered to youth living with HIV/AIDS could significantly improve and sustain the mean baseline adherence from 74.7% to 93.1% within 24 weeks (*p*< 0.001).

#### Secondary outcomes

Secondary outcomes reported in the included studies include immunologic and virologic outcomes, patient satisfaction with incoming messages, and quality of life.

**Immunologic and virologic outcomes**

**Fig 4:**
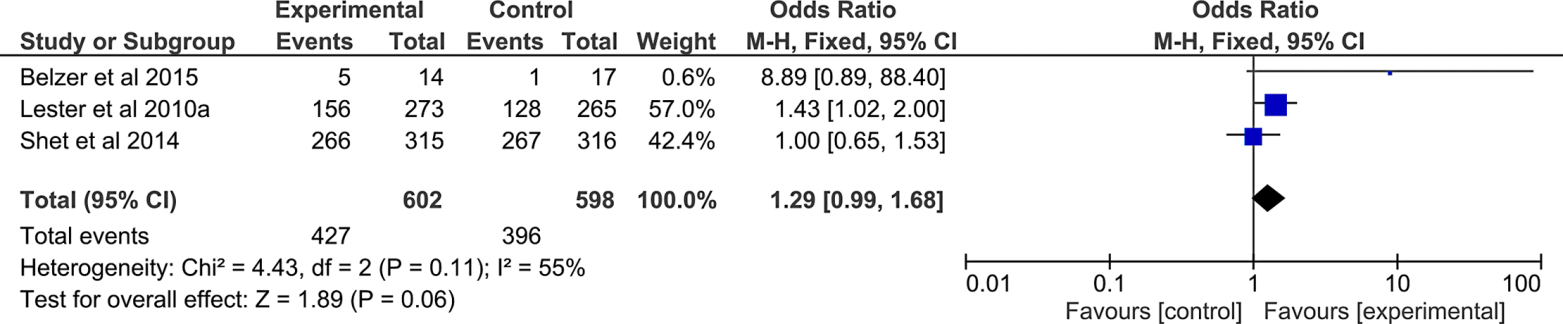
Forest plot of pooled odds ratio and 95% CI for effect of mobile phone intervention on viral load reduction.

Four (4) studies [23,45,50,51] involving 1194 participants (602 in intervention arm, 598 in control arm) were analysed. A positive effect of the mobile phone intervention on viral load was observed, but was not statistically significant (OR, 95%CI; 1.29, 0.99–1.68; p = 0.06), Fig 4.

**Participant experience with the mobile phone intervention (SMS-based only)**

Three (3) studies [46,48,52] provided data on how interventions were experienced by some participants. The majority rated the intervention as being useful in helping avoid missing doses, providing incentives for them to initiate their medications or maintain correct dosing schedule. Some participants further expressed a desire to continue receiving the SMS adherence messages even after the study [23,42,43] and were also willing to recommend the intervention to a friend [23,47]. Participants who did not find the intervention helpful cited timing between the arrival of SMS messages and actual medication intake being too far [42] or being at work or inconvenient place when messages arrived [43]. Adverse events reported include undelivered messages due to technical challenges [52] or lapse in cell phone service [42]; poor access to electricity to charge phones [24]; and stolen phones. A female participant in a study by Mbuagwa et al. [44] requested to withdraw from the study because she felt the intervention had compromised her undisclosed HIV positive status. Studies that employed mobile phone voice call functionality, however, did not report on how patients experienced the intervention.

Preference for voice or SMS as a means of message delivery was reported by Rodrigues et al. [53]. Of the 136 participants, 42 (34%), preferred only phone call, 15 (11%) preferred only SMS, 60 (44%) preferred both phone call and the SMS, while the rest preferred neither.

**Quality of life**

Quality of life was assessed with the WHOQOL-HIV BREF (World Health Organization Quality of Life in HIV-infected Persons instrument) [55] and the SF-12 QOL (Short From 12 Quality of Life) assessment form by Huang et al., [52] and Mbuagbaw et al., [47] respectively. Huang et al., [52] recorded a significant QOL (Quality of Life) improvements among treatment-naïve participants in the domains of physical health (*p* = 0.003), level of independence (*p* = 0.018), environment (*p =* 0.002), and spirituality/religion/personal beliefs (*p =* 0.021) whereas Mbuagbaw et al., [47] reported a mean quality of life score of 3.67 (SD-0.623) and 3.69 (SD-0.615) for participants in the SMS group and control groups respectively.

## Discussion

### Key findings

In this systematic review and meta-analysis, we investigated the effectiveness of mobile phone-based interventions for ART adherence and other outcomes among HIV positive individuals.

This review has four main findings. First, scheduled mobile phone SMS significantly improve adherence to ART among HIV positive patients. Triggered SMS did not have significant improvement in adherence to ART. Second, interventions delivered by voice call functionality did not improve adherence to ART. Third, current evidence from RCTs shows that mobile SMS interventions do not improve immunologic and virologic outcomes. However, A quasi-experimental study [44] also reports a statistically significant immunologic and virologic outcomes of SMS-based interventions. Finally, study participants expressed satisfaction with the mobile-phone-delivered ART adherence intervention.

### How the finding relates to existing literature

The current review updates and expands on the evidence by Hovarth et al., [21] and Finitsis et al., [22], who focused on SMS-based interventions and RCTs and concluded that SMS-based interventions are effective in improving adherence to ART. The current review supports this finding, and further reports that sending scheduled text messages (as opposed to real-time, triggered reminders) to individuals with HIV can significantly improve adherence. Our review additionally reports that mobile phone adherence interventions that allow for two-way communication may be more acceptable than standalone, SMS reminders. This finding corroborates existing literature that seems to suggest that stand-alone, SMS reminders may have a deteriorating effect on quality [56,57], as the reminders are seen to be intrusive and therefore produces habituation and response fatigue [22,46]. The current review identified design elements (such as directionality and the optimal frequency of intervention delivery) that might have moderated adherence outcomes. However, the extent to which these differences influenced adherence outcomes remains speculative, as most studies did not report on the theoretical constructs that informed their intervention design. Developing atheoretical mobile phone interventions may lead to a situation where any information, content or delivery methods could be included or excluded for unspecified reasons [58]. We, therefore, recommend adequate reporting of theories that inform intervention design in future studies.

Individual studies employing voice call as an intervention in this review have shown the potential of being effective in improving adherence, even though the pooled effect in a meta-analysis was not significant. This review, however, points to some beneficial effects, albeit secondary, that if harnessed, can improve patient experience of the mobile phone intervention. For example, a study by Huang et al. [52] in China found voice calls to be effective in enhancing the quality of life of individuals receiving ART. This finding contradicts earlier finding that standalone mobile phone text messaging leads to habituation and decrease in quality of life [22,46]. Additionally, adherence interventions delivered by voice calls may be more acceptable compared to text messaging as demonstrated by Rodrigues et al. [53]. Individuals likely to show a high preference for voice calls are aged and the illiterate population.

### Unanswered questions, future research and real-world implementation

More than half of the included studies (67%) used stand-alone, scheduled SMS reminders as an intervention to improve adherence. Beyond the research settings, little is known about how this intervention will be scaled up in low-literate populations and even among the aged [59] as SMS interventions will require these participants to read and respond to messages. Further exploration of the most suitable mobile phone functionality (SMS, Voice and IVR) is therefore warranted. It is also imperative that the World Health Organisation carefully review the current recommendation that SMS-based interventions, in general, are effective and instead focus on those interventions shown to be effective such as two-way text messages in their guidelines. Specific properties of mobile phone functionalities that can be tailored to specific populations such as the low literate group or the youth must be studied to provide specific recommendations to such groups.

Another finding of this review that can potentially impact scalability is the issue of phone ownership [59,60]. Access to a personal phone as reported in this review was the second most cited inclusion criteria. Lester et al., [23] for example excluded 49 (37%) of study participants for not having access to personal phones. Four (4) studies provided study phones [24,45,50,51]. It was however not clear how participants experienced the carrying of unfamiliar phones with different patterns of usage. We do not know in what situations cell phones may be provided and how this will affect cost and sustainability, or how we can determine who should be given a phone. These are practical issues that must be addressed if the intervention is to be scaled up in resource-constraint settings. Closely related to phone ownership is the issue of privacy. Even though our review points to practical steps adopted by individual studies to protect the undisclosed HIV status among study participants, privacy is still a potential challenge in using mobile phone to deliver care to HIV patients especially in developing countries. Mbuagbaw et al., [47] for example, reported that a patient withdrew from a study because of privacy concerns. This aspect of the intervention must be carefully considered on a scale up. Similarly, logistical concerns such as undelivered messages, broken phones and poor mobile network as reported in this review warrant attention. The review points to potential risks and unintended consequences associated with using mobile phones for adherence interventions and must be explored further.

Using a combination of weekly IVRs and neutral SMS reminders, Rodrigues and colleagues [53] have demonstrated significant improvement in adherence outcomes for patients in India, a setting characterised by illiteracy and multiple dialects. Given the lower use of SMSs in this setting, the study participants showed a preference for voice calls over SMS. A recent trial that looked at a combined mobile phone SMS and telephone call reminder (SMS reminder 3 days prior to scheduled clinic appointments and an average of 90sec lunch hour telephone call reminders a day prior to scheduled clinic appointment) and peer counselling found significant improvement in adherence and treatment outcomes among HIV positive patients on ART in Malaysia [61]. Acceptability of this intervention was high among the participants. Further exploration of combined functionality especially in resource-constraint settings is recommended.

Even though the focus of this review is not on SMART phones, it is important to state that new mobile messaging services such as Facebook Messenger, WhatsApp, SnapChat and Instagram, all have functions that are parallel and often surpass SMS and MMS functionality [62]. We expect that this intervention and other smartphone mobile phone interventions will become a more frequent feature for mobile phone adherence interventions for ART, especially in a high literate population and high-income countries. A study excluded from this review for example [63] found that augmented smartphone application that provided real-time information about the level of medication in the client’s blood and a pictorial depiction of immunoprotection significantly improved adherence to ART compared to the standard. We suggest further studies to look into how smartphone applications could be used to improve adherence to ART.

### Strength and limitations of the review

This is a comprehensive review of both RCTs and quasi-experimental interventions delivered by mobile phones to improve adherence to ART and other outcomes. Our review has several strengths. The adoption of a broad selection criterion to include all mobile phone-based interventions may reduce inflation of overall effect sizes as a way of preventing publication bias. The methods employed are reproducible, screening of trials and data extraction were carried out by two authors, with disagreement settled with a third reviewer through discussion.

There are however some limitations to our review. This relates to the wider scope of the review and the inclusion of studies without a control group. This might have introduced some confounding and selection bias respectively. The variation in interventions, outcome measurements and follow-up times might also have influenced the pooled effect size of the intervention. However, in situations where there was more than one follow-up, we included data on the longest follow-ups from the various studies.

## Conclusions

Scheduled mobile phone text-messaging interventions have demonstrated significant improvement in adherence to ART. Mobile SMS adherence interventions that allow for two-way communication may however be more acceptable than standalone SMS reminders, which are seen to be intrusive and therefore produces habituation and response fatigue. Mobile phone ownership is also a concern that needs to be addressed to enhance scalability in low and middle-income settings as this could affect the affordability and cost-effectiveness of the intervention. Voice calls and triggered SMS functionalities do not have a significant effect on adherence to ART although there is a higher preference for voice functionality over SMS in limited-resource and low-literacy settings. Further exploration of the mobile voice functionality and its possible combination with scheduled SMS is recommended. Evidence provided in this study will guide the implementation of the mobile phone intervention to improve adherence to ART among HIV positive patients, by addressing practical challenges that could militate against scalability and roll out especially in limited resource settings.

## Supporting information

S1 File PRISMA 2009 checklist. (DOC)

S2 File Results of risk of bias assessment. (DOCX)

S1 Table Search strategy for PUBMED. This search strategy was modified across databases as appropriate.
(DOCX)
