# Supplementary material for: Effectiveness of short message services and voice call interventions for antiretroviral therapy adherence and other outcomes: A systematic review and meta-analysis
Source: PLoS One. 2018 Sep 21;13(9):e0204091. doi: 10.1371/journal.pone.0204091 (PMC6150661; doi:10.1371/journal.pone.0204091)
Supplement: S2 File — (DOCX) [file pone.0204091.s002.docx]

**Table A: Risk of bias of included RCTs**

| **Author (year**) | Participant blind to treatment assignment | Adequate blinding of participants | Adequate allocation concealment | Description of drop out and withdrawal | Outcome assessors blinded to treatment allocation | Groups similar at baseline | Identical treatment of two groups | Similar measure of outcome for both group | Were outcomes measured in the same way | Were outcome  measured in a reliable way? | Were statistics relevant | Those delivering treatment blind | Appropriate trial design | Total |
| --- | --- | --- | --- | --- | --- | --- | --- | --- | --- | --- | --- | --- | --- | --- |
| **da Costa et al. (2012)** | N | N | Y | Y | Y | Y | Y | Y | Y | Y | Y | Y | Y | 11/13 |
| **Huang et al 2013** | N | U | Y | U | U | Y | Y | Y | Y | Y | Y | U | Y | 8/13 |
| **Kalichman et al. (2011)** | Y | U | U | U | Y | U | N | Y | Y | Y | Y | Y | Y | 8/13 |
| **Lester et al. (2010)** | N | N | Y | Y | Y | Y | Y | Y | Y | Y | Y | N | U | 9/13 |
| **Mbuagbaw et al (2012)** | N | N | Y | Y | Y | Y | Y | Y | Y | Y | Y | Y | Y | 11/13 |
| **Pop-Eleches et al. (2011)** | N | N | U | Y | U | Y | Y | Y | Y | Y | Y | U | U | 7/13 |
| **Maduka & Tobin-West (2012)** | Y | Y | Y | Y | U | Y | Y | Y | Y | Y | Y | Y | Y | 12/13 |
| **Shet et al**  **(2014)** | N | N | Y | Y | Y | Y | Y | Y | Y | Y | Y | Y | Y | 11/13 |
| **Belzel et al (2014)** | N | U | Y | Y | U | U | N | Y | Y | Y | Y | N | Y | 7/13 |
| **Sabin et al (2015)** | N | N | Y | Y | N | Y | Y | Y | Y | Y | Y | N | Y | 9/13 |
| **Harberer**  **(2016)** | Y | Y | U | Y | U | Y | Y | Y | Y | Y | Y | N | Y | 10/13 |

**Table B: Risk of bias across cohort/quasi-experimental studies**

| Author (year) | Sample representative of population | Patients at similar point of illness/breakfast | Selection biased been dealt with | Confounding factors identified and dealt with | Are outcomes assessed using objective criterion | Was follow up carried out over a sufficient period | Description of drop out and withdrawal | Reliable measure of outcome | Was appropriate statistical analysis used | Total | |
| --- | --- | --- | --- | --- | --- | --- | --- | --- | --- | --- | --- |
| **Rodrigues et al. (2012)** | Y | N | Y | U | Y | Y | U | Y | Y | 6/9 |  |
| **Lewis et al (2013)** | Y | Y | U | U | N | Y | Y | Y | Y | 6/9 |  |
