# Supplementary material for: Effectiveness of short message services and voice call interventions for antiretroviral therapy adherence and other outcomes: A systematic review and meta-analysis
Source: PLoS One. 2018 Sep 21;13(9):e0204091. doi: 10.1371/journal.pone.0204091 (PMC6150661; doi:10.1371/journal.pone.0204091)
Supplement: S1 Table — This search strategy was modified across databases as appropriate. (DOCX) [file pone.0204091.s003.docx]

| **Search** | **Database** | **Hits** |
| --- | --- | --- |
|  | **Pubmed** |  |
| **#4** | **"Search ((#1) AND (#2) AND (#3))** | **825** |
| **#3** | #3,"Search (HIV infections[MeSH Terms]) OR HIV[MeSH Terms]) OR hiv[Title/Abstract]) OR hiv-1[Title/Abstract]) OR hiv-2*[Title/Abstract]) OR hiv1[Title/Abstract]) OR hiv2[Title/Abstract]) OR hiv infect*[Title/Abstract]) OR human immunodeficiency virus[Title/Abstract]) OR human immune deficiency virus[Title/Abstract]) OR human immunodeficiency-deficiency virus[Title/Abstract]) OR human immune-deficiency virus[Title/Abstract]) OR human immun*[Title/Abstract]) AND deficiency virus[Title/Abstract]) OR acquired immunodeficiency syndromes[Title/Abstract]) OR acquired immune deficiency syndrome[Title/Abstract]) OR acquired immuno-deficiency syndrome[Title/Abstract]) OR sexually transmitted diseases, viral[MeSH Terms]) AND HIV[Title/Abstract]) OR HIV/AIDS[Title/Abstract]) OR HIV-infected[MeSH Terms]) OR HIV[Title]) OR HIV/AIDS[Title]) OR HIV-infected[Title]",227529,21:59:53 | **227529** |
| **#2** | #2,"Search (randomized controlled trial[Publication Type]) OR controlled clinical trial[Publication Type]) OR randomised controlled trial[MeSH Terms]) OR random allocation[MeSH Terms]) OR double-blind method[MeSH Terms]) OR single-blind method[MeSH Terms]) OR clinical trial[Publication Type]) OR trial[Text Word]) OR clinical trials[MeSH Terms]) OR clinical trial[Text Word]) OR singl*[Text Word]) OR doubl*[Text Word]) OR trebl*[Text Word]) OR tripl*[Text Word]) AND mask*[Text Word]) OR blind*[Text Word]) OR placebos[MeSH Terms]) OR placebo*[Text Word]) OR random*[Text Word]) OR non-random*[Text Word]) OR research design[MeSH Subheading]) OR follow-up studies[MeSH Terms]) OR prospective studies[MeSH Terms]) OR control*[Text Word]) OR prospectiv*[Text Word]) OR volunteer*[Text Word]) OR longitud*[Text Word]) OR descripti*[Title/Abstract]) OR study[Title/Abstract]) OR quasi-experimental[MeSH Terms]) OR evaluat*[Title/Abstract]",11211789,21:26:58 | **11211789** |
| **#1** | #1,"Search (medication adherence[MeSH Terms]) AND cellular phone*[MeSH Terms]) OR Adherence[Title]) OR compliance[Title]) AND telephone[Title/Abstract]) OR mobile[Title/Abstract]) OR cellphone[Title/Abstract]) OR sms [Title/Abstract]) OR voice[Title/Abstract]) OR text*[Title]) OR intermittent voice response[Title/Abstract]) OR IVR[Title/Abstract]",129891,20:51:55 | **129891** |
| Filters | Publication date from 1995/01/01 to 2017/12/31;  Humans",825,22:08:27 |  |
